# Antimicrobial Activity of Cathelicidin-Derived Peptide from the Iberian Mole *Talpa occidentalis*

**DOI:** 10.3390/vaccines10071105

**Published:** 2022-07-10

**Authors:** Andrea Otazo-Pérez, Patricia Asensio-Calavia, Sergio González-Acosta, Victoria Baca-González, Manuel R. López, Antonio Morales-delaNuez, José Manuel Pérez de la Lastra

**Affiliations:** 1 Biotechnology of Macromolecules Research Group, Instituto de Productos Naturales y Agrobiología (IPNA-CSIC), Avda. Astrofísico Francisco Sánchez, 3, 38206 San Cristóbal de la Laguna, Spain; andreaotazopz@gmail.com (A.O.-P.); patriciaac@ipna.csic.es (P.A.-C.); sergi_glez@hotmail.com (S.G.-A.); victoria@ipna.csic.es (V.B.-G.); lolo0707mrl@gmail.com (M.R.L.); morales.delanuez@ipna.csic.es (A.M.-d.); 2 Escuela de Doctorado y Estudios de Posgrado, Universidad de La Laguna, Avda. Astrofísico Francisco Sánchez, SN. Edificio Calabaza-Apdo. 456, 38200 San Cristóbal de La Laguna, Spain

**Keywords:** *Eulipotyphla*, insectivores, mammals: innate immunity, antimicrobial peptide, *Talpidae*

## Abstract

The immune systems of all vertebrates contain cathelicidins, a family of antimicrobial peptides. Cathelicidins are a type of innate immune effector that have a number of biological functions, including a well-known direct antibacterial action and immunomodulatory function. In search of new templates for antimicrobial peptide discovery, we have identified and characterized the cathelicidin of the small mammal *Talpa occidentalis*. We describe the heterogeneity of cathelicidin in the order *Eulipotyphla* in relation to the Iberian mole and predict its antibacterial activity using bioinformatics tools. In an effort to correlate these findings, we derived the putative active peptide and performed in vitro hemolysis and antimicrobial activity assays, confirming that Iberian mole cathelicidins are antimicrobial. Our results showed that the Iberian mole putative peptide, named To-KL37 (KLFGKVGNLLQKGWQKIKNIGRRIKDFFRNIRPMQEA) has antibacterial and antifungal activity. Understanding the antimicrobial defense of insectivores may help scientists prevent the spread of pathogens to humans. We hope that this study can also provide new, effective antibacterial peptides for future drug development.

## 1. Introduction

Antimicrobial peptides (AMPs) are a broad category of gene-encoded short peptides that have been identified in almost all life forms. AMPs are small molecules (between 12 and 150 amino acids) that are usually cationic in nature [1,2]. Because of their positive charge, AMPs have an affinity for the membranes of bacteria, which are often negatively charged [3]. In addition, AMPs serve crucial functions in the host’s innate immune response to microbial infections [4]. Typically, the hydrophilic amino acids of most AMPs are separated from the hydrophobic ones, which provides them the capacity to permeate the amphipathic membrane of bacteria [5]. Cathelicidins are a family of AMPs found in the immune systems of all vertebrates. Initially found in bovine neutrophils, cathelicidins are important components of the host’s innate immunological response against bacterial infection [6]. Most cathelicidins, like other AMPs, have direct killing action against a wide range of microbes and are actively implicated in many stages of host immune regulation and disease resistance [7]. The gene structures of cathelicidins from several vertebrate species appear to be relatively constant, although the mature peptides expressed are quite diverse, particularly for mammals. The tremendous diversity in AMP genes across evolution may have coincided with adaptations to the exposure to their environmental microbiota. This emphasizes the importance of studying cathelicidins and the rest of the AMPs in species biodiversity, as they may contain an immune system that is specifically adapted for different types of bacteria, depending on the microorganisms with which they have co-evolved in their ecological niche [8].

Among placental mammals, insectivores are considered the most primitive and are widely regarded as the ancestors of present-day mammals. The insectivore group makes an important contribution to the environment by suppressing insects and pests [9]. The Iberian mole (*Talpa occidentalis*) is a soricomorphic mammal in the *Talpidae* family that feeds on subterranean larvae and worms. It is mainly found in Spain and Portugal and it belongs to the *Eulipotyphla* order [10]. *Eulipotyphla* is the third most diverse order of mammals, with more than 450 identified extant species. *Eulipotypha* comprises little mammals with long, pointed snouts that measure 8 to 30 cm in length. They are solitary animals that are most active at night, however there are some that are active during the day. All of them excavate tunnels or holes to protect themselves from predators [11].

As a class of essential effectors in the innate immune system, cathelicidins contain a variety of biological functions that include a well-known direct antibacterial action and immunomodulatory function [7,12]. In order to confirm the antimicrobial activity of cathelicidins from Iberian moles and to explore other templates for antimicrobial peptide discovery, we here describe the identification and characterization of the *Talpa occidentalis* cathelicidin. We study the diversity of cathelicidin in the *Eulipotyphla* order as it relates to the Iberian mole, and use bioinformatic tools for the structural analysis and prediction of its antimicrobial properties. In an attempt to correlate these predictions, we chemically derived the putative active peptide and performed some in vitro assays for hemolysis and antimicrobial activity.

## 2. Materials and Methods

### 2.1. Retrieval of Iberian Mole Cathelicidin Sequences

The annotated sequences of cathelicidins from the Iberian mole (*Talpa occidentalis*) XP_037384309 (isoform X1) and XP_037384317 (isoform X2) were retrieved from the protein database at the National Center for Biotechnology Information (NCBI) in June 2021. The sequences were downloaded in FASTA format for further analysis. According to the information from this database, these sequences were derived by automated computational analysis using the gene prediction method Gnomon [13]. Supporting evidence includes similarity to 100% coverage of the annotated genomic feature by RNAseq alignments, including two samples with support for all annotated introns.

The amino acid sequences were verified as belonging to the cathelicidin family by identifying the cathelin conserved domain with the NCBI “Identify Conserved Domains with CD-search” tool [14]. This server uses “reverse position-specific BLAST” to compare a query protein sequence against conserved domain models collected from a number of source databases, and presents the results as a concise display. If CD-Search finds a specific hit, there is high confidence in the association between the protein query sequence and a conserved domain, also resulting in a high confidence level for the inferred function of the protein query sequence.

The nucleotide sequence of the gene and the corresponding exon/intron organization was obtained from the LOC119259496 gene accession of NCBI.

The elastase site on the amino acid sequence of the fourth exon of the Iberian mole cathelicidin was predicted by using the “PeptideCutter” tool from the Expasy web site [15] (https://web.expasy.org/peptide_cutter/ accessed on 9 May 2022). The signal peptide was predicted by SignalP 6.0 [16]. The amino acid sequence of the human cathelicidin peptide LL -37 was used as a reference for the comparative bioinformatic analysis of cathelicidins.

### 2.2. In Silico Analyses of Physicochemical Properties of Peptides

The isoelectric point, net charge, molecular weight and Boman index was predicted using the “Peptide Property Calculator” web service (Lear, Cobb L., 2016). Hydrophobicity of the peptide was predicted by the “Peptide 2.0” web service [Peptide Hydrophobicity/Hydrophilicity Analysis Tool (peptide2.com)].

### 2.3. Multiple Alignment and Phylogenetic Tree

The multiple sequence alignment of *Eulipotyphla* cathelicidins was performed using the Constraint-based Multiple Alignment Tool (COBALT). COBALT is a multiple sequence alignment tool that finds a collection of pairwise constraints derived from conserved domain database, protein motif database, and sequence similarity, using RPS-BLAST, BLASTP, and PHI-BLAST [17]

For the phylogenetic analysis of the *Eulipotyphla* cathelicidins, we used the exons 1-3 for each cathelicidin available at the NCBI database on May 2022, with the following accession numbers: *Galemys pyrenaicus* (KAG8525175), *Erinaceus europaeus* isoform X1 (XP_007533667) *Erinaceus europaeus* isoform X2 (XP_016048219), *Sorex araneus* (XP_004615204), *Condylura cristata* (XP_012590677), *Talpa occidentalis* isoform X1 (XP_037384309), and *Talpa occidentalis* isoform X2 (XP_037384317). The NCBI COBALT tool computes a multiple protein sequence alignment using conserved domain and local sequence similarity information. Using this alignment, the phylogenetic tree was constructed with the COBALT tree tool from given distances (or dissimilarities) between sequences using the Fast Minimum Evolution algorithm [18]. The maximum allowed fraction of mismatched bases in the aligned region between any pair of sequences was of 0.85. The Grishin method was used for the calculation of the evolutionary distance between the two sequences modeled [19]. The COBALT alignment was performed with default parameters.

### 2.4. In Silico Analysis of Biological Activity

The predicted biological activity of peptides against different types of microorganisms was obtain using several online servers (accessed on May 2022). For antimicrobial activity we used the server CAMPR3 (http://www.camp.bicnirrh.res.in/prediction.php accessed on 9 May 2022) with the prediction option “Support Vector Machine” (SVM) [20,21]. Antiviral characteristics of peptides were predicted using AVPpred (http://crdd.osdd.net/servers/avppred/ accessed on 9 May 2022), which used an antiviral peptides prediction algorithm created utilizing peptides having experimentally demonstrated antiviral activity [22]. The prediction of activity against specific microbial species and human erythrocytes was performed using the Database of Antimicrobial Activity and Structure of Peptides (DBAASP) web server (https://dbaasp.org/home accessed on 9 May 2022) [23]. The prediction of antifungal activity was performed by the Antifp server (https://webs.iiitd.edu.in/raghava/antifp/index.html accessed on 9 May 2022). The anticancer activity was determined by the sequence-based predictor iACP-FSCM [24].

### 2.5. Structural Analysis of Peptides

The helicity region of the peptides was analyzed by the service HeliQuest (https://heliquest.ipmc.cnrs.fr/ accessed on 9 May 2022), which also provided a visual representation of the results. The algorithm examines whether a segment contains an uninterrupted hydrophobic face, that is it contains at least five hydrophobic residues that are adjacent when represented on a helical wheel. If a hydrophobic face exists, the procedure examines whether the facing residues are polar or poorly hydrophobic. Heliquest contains a decision tree that combines results from TMHMM [25,26] and PSIPRED [27] with a discriminant factor based on an analysis of lipid-biding helices. This server was also able to calculate the hydrophobic moment, denoted by the symbol ‘H’ and ranging from 0 to 3.26. A high value for <H> indicates that the helix is amphipathic in the direction that is perpendicular to its axis.

### 2.6. Modeling of Peptides

The model of the Iberian mole cathelicidin active peptide was computed by the Alphafold server homology modeling pipeline [28]. ColabFold sends a FASTA input sequence to an MMseqs2 server, which searches two databases, UniRef100 and a database of environmental sequences, with three profile-search iterations each. The input for the search of the second database is a sequence profile that was produced by the first search of the UniRef100 database. The server generates two A3M-formatted MSAs comprising all sequences that have been identified. The models ranked in the first position were considered in the analysis. For the visualization of the peptide 3D-structures we used ChimeraX software [29].

For the interaction of peptides with membranes, we utilized the PPM server (https://opm.phar.umich.edu/ppm accessed on 9 May 2022), which determines the rotational and translational locations of transmembrane and peripheral proteins in membranes given their 3D structure [30,31].

### 2.7. Molecular Docking Studies with TLR4/MD2 Complex

The Iberian mole cathelicidin active peptide was subject to a molecular docking study. The crystallographic structure of the TLR4/MD2 complex was obtained from the PDB bank (PDB code: 4G8A), and visualized using ChimeraX. The complex structure of TLR4/MD2-with the Iberian mole cathelicidin active peptide was accomplished by GalaxyTongDock, and performs ab initio rigid-body docking to predict the complex structure of two proteins. For the analysis, the plausible docking model with the lowest interface binding energy and higher TongDock_A score was chosen [32].

### 2.8. Peptide Synthesis

Peptides were synthesized at a 10 mg scale (Caslo, Lyngby, Denmark) with a purity of greater than 90% (analyzed by HPLC). The N-terminal end of peptides was acetylated and the C-terminal end was amidated to prevent their degradation during processing. Aliquots of each polypeptide (2 mg) were diluted in distilled water at a stock concentration of 1 mM to facilitate assay efficiency.

### 2.9. Antimicrobial Activity In Vitro

We used the minimum inhibitory concentration (MIC) assay [33] to obtain the minimum concentration for each peptide that was able to inhibit the microbial growth. The microorganisms used were *Escherichia coli* (CECT 434), *Staphylococcus aureus* (CECT 794), *Campylobacter jejuni* (CECT 9112), *Salmonella enterica* (CECT 456), *Pseudomonas aeruginosa* (CECT 108) and *Candida albicans* (CECT 1392), since they are good representatives of the Gram negatives and Gram-positive bacteria and yeast groups respectively. The bacteria were first cultured on Mueller Hinton agar media and the yeast on Sabouraud media at 37 °C for 24 h. Subsequently, the isolated colonies were transferred to broth media and incubated at 37 °C for 16 h. In a 96-well plate, serial dilutions of peptides were prepared to obtain final concentrations from 50 µM to 0.1 µM. The inocula were prepared at 0.5 McFarland, equivalent to 1.5 × 10^8^ cells/mL, in broth media. For the negative control we used the sterile culture media and the microorganism without peptide as a growth control. The MIC was calculated using the OD values at 595 nm at 18 h of incubation. For kinetic studies, the microtiter plate was incubated at 37 °C for 18 h, A_595_ was measured at one-hour intervals, and a graph of OD was plotted against time. The procedure was performed in triplicate.

### 2.10. Hemolytic Activity

The hemolytic activity of the peptide was tested in vitro. For this purpose, washed red blood cells (Deltaclon, Madrid, Spain) were tested in a 96-well plate against a serial dilution of peptides. The amount of free hemoglobin produced by hemolysis was determined by measuring the absorbance at 405 nm after 45 min of incubation at 37 °C. Triton X detergent was used as lysis control, and saline solution was used as negative control. To analyze the effect of peptide concentration on hemolysis rate, a parametric one-way ANOVA was used. A Tukey’s test was performed to determine the differences between the mean values.

## 3. Results

**Figure 1:**
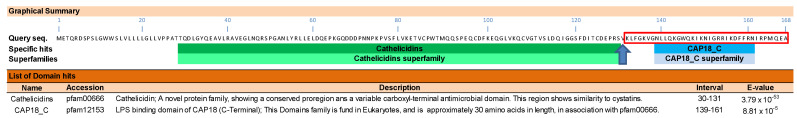
Graphical analysis of *Talpa occidentalis* cathelicidin isoform X1 (XP_037384309) when subjected to the “Identify Conserved domains” analysis tool of the NCBI protein database. The exact interval range and score (E-value) of each domain hits (pfam00666 and pfam12153) are shown in the “List of domain hits” table (below). The vertical blue solid arrow indicates the predicted position for the elastase cleaving site. The sequence of the antimicrobial portion of the putative peptide for the cathelicidin of *Talpa occidentalis* is within the red box.

Examination of the annotated sequences of two isoforms of *Talpa occidentalis*, cathelicidin X1 and X2, revealed that both isoforms were differing in one amino acid. The glutamine 67 of the X1 isoform (XP_037384309), which was missing in the isoform X2 (XP_037384317). All cathelicidins have an N-terminal pro-sequence of approximately 100 residues followed by a more diverse antimicrobial portion [34,35]. When subjected to the analysis tool “Identify Conserved domains” of the National Center for Biotechnology Information (NCBI) protein database, both isoforms were distinguished by a highly conserved cathelin-like precursor sequence located at their N-terminal region (from position 30 to 131 of isoform X1). This region showed similarity with cystatins and was identified for homology with the pfam00666 domain from the protein family (pfam) database and a more diverse region (positions 139 to 161 of the X1 isoform) that corresponded to the mature AMP. This region contained the LPS binding domain of CAP18 (pfam12153), which is often found in eukaryotes in association with pfam00666. For the isoform X1, the corresponding E-value of the similarity with the pfam00666 and pfam12153 was of 3.79 × 10^−53^ and 8.813 × 10^−5^, respectively (Figure 1).

**Figure 2:**
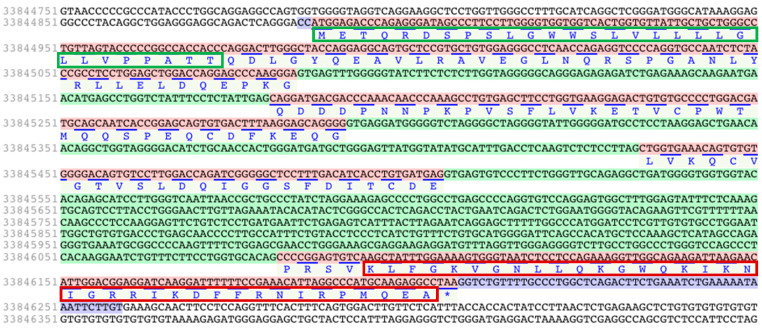
Nucleotide and amino acid sequence of the gene coding for the X1 variant of the Iberian mole (*Talpa accidentalis)*. The nucleotide sequence of the introns is shaded in green and the exons are in red. The translated sequence of the exons is in blue. The mRNA ends of the transcript variant X1 (1375 bp) is shaded in purple. The elastase cleaving site was predicted for the first valine or alanine of the amino acid sequence from the fourth exon. The sequence of the signal peptide is within the green box. The sequence of the antimicrobial portion of the putative peptide for the cathelicidin of *Talpa occidentalis* is within the red box. The figure was a capture of the NCBI Genome Data Viewer using the tool “Sequence text view”.

Similar to other cathelicidins, the genomic organization of *Talpa occidentalis* cathelicidin gene consisted of four exons. Exons 1-3 were coding for the signal peptide and cathelin-like domain, whereas exon 4 was coding for the diverse portion corresponding to the active antimicrobial peptide (Figure 2).

**Figure 3:**
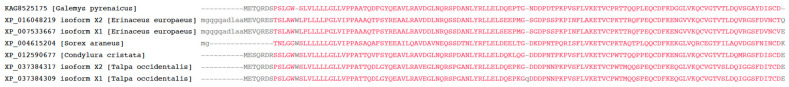
Alignment performed with the Constraint-based alignment COBALT [17] tool of the amino acid sequence of exons 1-3 of NBCI-annotated cathelicidins (accessed on May 2022) from the *Eulipotyphla* order. Sequences comprise the Iberian desman (*Galemys pyrenaicus*), the European hedgehog (*Erinaceus europaeus*), the common shrew (*Solex araneus*), the star-nosed mole (*Condylura cristata*) and the Iberian mole (*Talpa occidentalis*). Identical amino acids among all sequences are highlighted in red.

For the study of the evolutionary relationship of *Talpa occidentalis* cathelicidin with other mammals from the *Eulipotyphla* order, we retrieved the protein sequence from the NBCI database corresponding to exons 1-3 of the Iberian desman (*Galemys pyrenaicus*), the European hedgehog (*Erinaceus europaeus*), the common shrew (*Solex araneus*), and the star-nosed mole (*Condylura cristata*). The COBALT alignment of this region showed a high degree of conservation. The main differences among the sequences of the *Eulipotyphla* order were observed at the N-terminal signal peptide, and at the last amino acid located at the end of the exon 3 (Figure 3).

**Figure 4:**
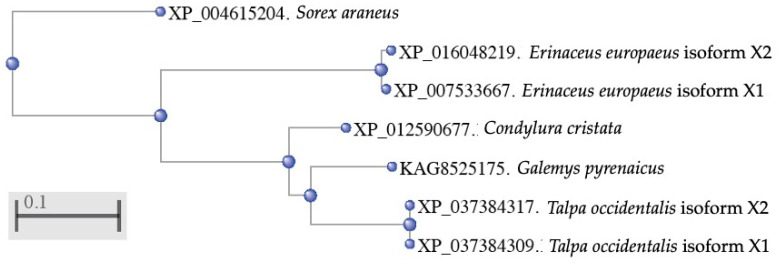
Phylogenetic tree constructed using alignment of the exons 1-3 of annotated cathelicidins from the *Eulipotyphla* order. Multiple sequences comprised the Iberian desman (*Galemys pyrenaicus*), the European hedgehog (*Erinaceus europaeus*), the common shrew (*Solex araneus*), the star-nosed mole (*Condylura cristata*) and the Iberian mole (*Talpa occidentalis*). The phylogenetic tree was constructed using the COBALT tool [17], from the NCBI, with the Fast Minimum Evolution algorithm.

In order to study the evolutionary relationship of *Eulipotyphla* cathelicidins comprising exon 1-3, we next constructed a phylogenetic tree of the aligned sequences using the COBALT tree tool of the NCBI. We observed that the *Eulipotyphla* sequences formed distinct groups. The bottom cluster of the tree comprised the sequences of the two isoforms identified in the Iberian mole (*Talpa occidentalis*). These two sequences were in close proximity with the sequence of the Iberian desman (*Galemys pyrenaucus*), which, together with the sequence of the star-nosed mole (*Condylura cristata*), formed a separate cluster. The two sequences of the European hedgehog (*Erinaceus europaeus*) corresponding to the cathelicidin isoforms X1 and X2 formed a distinct cluster. Finally, the cathelicidin sequence of the common shrew (*Solex araneus*), formed a separated branch, suggesting that this cathelicidin gene could have originated from a common ancestor (Figure 4).

When the *Talpa occidentalis* cathelicidin precursor was analyzed, it was shown to have the same cleavage site as most cathelicidins [36]. Hence, the site for elastase cutting site of the putative peptide was predicted for the first valine or alanine of the amino acid sequence from the fourth exon. In this case it corresponded to the valine 131 of the isoform X1 from the Iberian mole cathelicidin. We named the mature antimicrobial peptide of the Iberian mole cathelicidin as “To-KL37”, with the first two letters standing for the initial of the genus and species, corresponding; followed by the first two amino acids letters of the peptide and the number of amino acids in its sequence. Similarly, we predicted and named the putative antimicrobial peptide for the annotated cathelicidins of all members of the *Eulipotyphla* order (Table 1), with the exception of the Iberian desman (*Galemys pyrenaicus*), which lacked the fourth exon in its annotated sequence.

**Figure 5:**
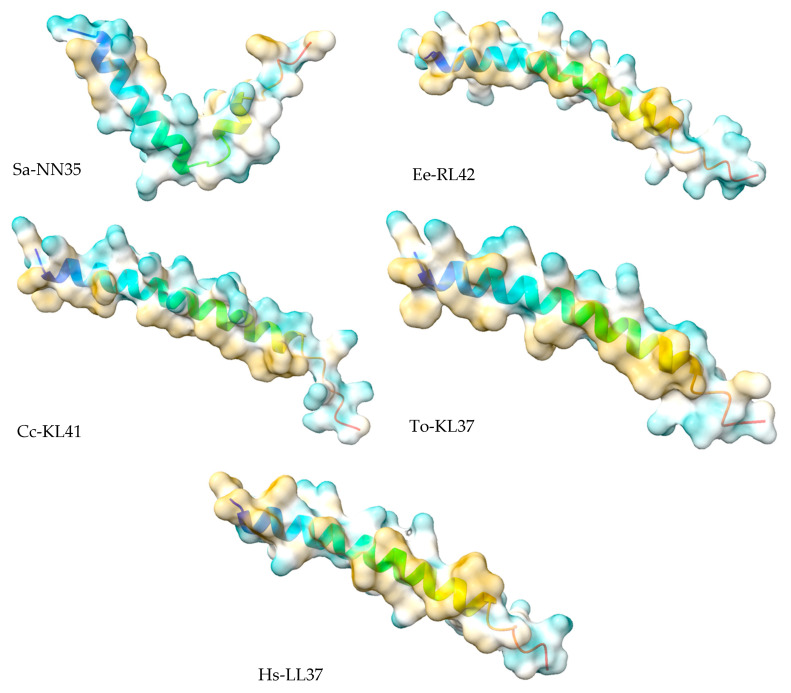
Alphafold structural models of putative antimicrobial peptides from the annotated cathelicidins of *Eulipotyphla* order, including *Talpa occidentalis*. The human cathelicidin peptide LL37 was used as a reference. ChimeraX software was used for the visualization of the structures. The tertiary structure of the peptide is shown in combination with the hydrophobicity as a transparent surface. All models used were those ranked-1 by the Alphafold server. The results are shown with coloring on the molecular surface ranging from dark cyan (most hydrophilic) to white to dark goldenrod (most lipophilic).

We next modelled the putative *Eulipotyphla* peptides in order to elucidate their secondary and tertiary structures. This will help us to investigate the potential for interaction with the biological membranes and other structural features that contribute to the antimicrobial activity. For comparison with the physicochemical properties of a well-known cathelicidin, we also modelled the human cathelicidin Hs-LL37 (LLGDFFRKSKEKIGKEFKRIVQRIKDFLRNLVPRTES) and used it for structural studies as a peptide of reference for cathelicidin-derived AMPs (Figure 5). In the same way, all predicted peptides of this order, except Sa-NN35, had a CAP18 region (pfam12153, Table 1).

According to the predicted models, the secondary structure of all predicted models for the active peptide comprised an alpha-helical portion between 30 and 34 amino acids. The only exception was the common shrew (*Solex araneus*), which showed a tertiary structure of two non-linear alpha helical stretches of 19 and 6 residues (Figure 5) separated by three residues (DPN).

**Figure 6:**
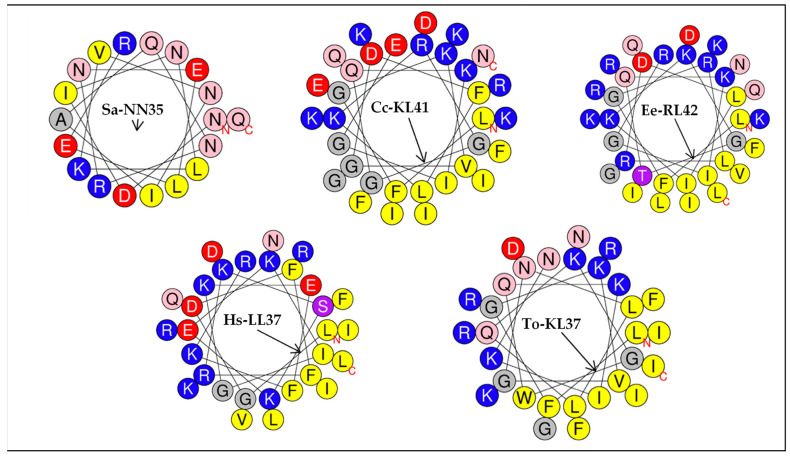
Helical wheel projection by Edmonson of alpha helices included in the putative antimicrobial peptides from the annotated cathelicidins of the *Eulipotyphla* order, encompassing *Talpa occidentalis*. Residues in yellow are hydrophobic, whereas residues colored in blue are cationic. Polar acidic residues (D and E) are colored in red, amine group-containing residues (N and Q) are colored in pink, hydroxi-amino acids (S and T) are colored in purple, and glycine residues are colored in grey. The arrow indicates the orientation of the hydrophobic moment <µH>. The human cathelicidin peptide Hs-LL37 was used as a reference.

In order to ascertain whether hydrophobic amino acids were concentrated on one side of the helix, with polar or hydrophilic amino acids on the other, we represented and computed the hydrophobicity and net charge of each peptide using the helical wheel projection by Edmonson, where alpha helices are represented by two dimensional projections (Figure 6).

The helical representation of this portion of the peptide allowed us to identify the hydrophobicity, net charge, hydrophobic moment and the hydrophobic face oriented toward the hydrophobic core of each peptide (Table 2).

Helical wheel analysis showed that putative sequences derived from *Eulipotyphla* cathelicidins formed distinct amphipathic structures and all showed a hydrophobic face with hydrophilic residues facing upward. According to this computation, with the exception of Sa-NN35, all alpha-helices were hydrophobic, with values between 0.174 (of Hs-LL37) and 0.338 (of To-KL37) and cationic, with a net positive charge between 5 (of Cc-KL41) to 9 (of Ee-RL42). The hydrophobic face of the common shrew Sa-NN35 (LLI) was the shortest of all *Eulipotyphla* helices. In contrast, the helix of the Iberian mole (To-KL37) showed the longest hydrophobic face (LIGIVIILFFGW) of all peptides studied. The lowest value for the hydrophobic moment (0.148) corresponded to the helix of the common shrew (*Solex araneus*) Sa-NN35.

We next studied the possible influence of the amphipathic properties of the helices and tertiary structures of the putative peptides of the cathelicidins from the *Eulipotyphla* order in the capacity to interact with biological membranes. For this purpose, we subjected the Alphafold peptide models to the OPM server, a computational tool for the study of the spatial arrangement of protein structures in lipid bilayers (Table 3).

Consistent with the peptide of reference, human cathelicidin Hs-LL-37, all putative cathelicidins from the *Eulipotyphla* order showed interaction with membranes displaying tilting angles between 80–90 degrees and a similar number of embedded residues (Table 3). The only exception was the peptide from the common shrew Sa-NN35, which displayed a distinct tilt angle of 67 degrees and an orientation with the membranes that resulted in the embedding of only three residues (at positions 33–35).

In order to investigate the possible correlation of the different structures of *Eulipotyphla* cathelicidin with their antimicrobial activity, we subjected all sequences to bioinformatic tools for the prediction of their antimicrobial, antifungal and antiviral activities (Table 4).

According to these computational tools, none of the peptides were predicted to have anti-fungal activity. The Iberian mole peptide To-KL37 obtained the highest value for the prediction of the antimicrobial activity of all peptide studied (with a value of 0.946), followed by the peptide Ee-RL42 (0.902). The human cathelicidin Hs-LL37 used as a peptide of reference obtained a score of 0.762 using this bioinformatic tool for the prediction of its antimicrobial activity. The lowest value for the predicted antimicrobial activity (0.54) corresponded to the peptide from common shrew (Sa-NN35).

Concerning the antiviral activity, the Iberian mole peptide (To-KL37) was also showed to have the highest percentage for the predictions of all peptides studied, with a value of 80.66%, close to the percentage obtained for the human cathelicidin Hs-LL37 (78.13%), used as a peptide of reference. According to this predictive tool, the peptide from the common shrew (Sa-NN35) was consider to having no antiviral activity.

We next subjected the *Eulipotyphla* peptides to the in silico analysis of the antimicrobial activity against specific microorganisms (Table 5).

From this analysis, none of the peptides were predicted to be active against *Candida albicans* and *Saccharomyces cerevisiae* (not shown). The putative peptide from the Common shrew Sa-NN35 showed no activity with any microorganisms studied, which was consistent with the lower value obtained from the previous analysis of its in silico antimicrobial activity (Table 4) and with the distinct tertiary structure, helical properties and mode of interaction with membranes. The peptides Ee-RL42 and Cc-KL41, from the European hedgehog and the star-nosed mole, respectively, were predicted to be active only against the microorganisms *E. coli* and *P. aeruginosa*. However, the peptide Cc-KL41 was predicted to be active against human erythrocytes. The peptide from the Iberian mole To-KL37 was predicted to be active against all microorganism studied, whereas the peptide of reference, human cathelicidin Hs-LL37, was predicted to be active against *E. coli* and *P. aeruginosa.*

In summary, the putative peptide To-KL37 had the largest hydrophobic face, greater hydrophobicity, and a higher score in predicted antimicrobial activity among the four small mammals studied. Therefore, To-KL37 was selected to further investigate the antimicrobial and hemolytic activity in vitro against various microorganisms and human erythrocytes, respectively. In our in vitro analysis of antimicrobial activity, To-KL37 exerted broad spectrum activity but moderate antimicrobial abilities, with MIC values ranging from 0.78 to 25 µM depending on the bacteria tested (Table 6).

According to this assay, the synthetic peptide To-KL37 was still active at concentrations of 1.56–0.78 µM when tested against *Campylobacter jejuni.* In contrast, this peptide was less active against *Pseudomonas aeruginosa*, requiring higher concentrations of 25–12.5 µM. Moderate concentrations of the peptide were necessary to inhibit the growing of the microorganisms *Staphylococcus aureus*, *Escherichia coli*, and *Salmonella enterica* (Table 6).

**Figure 7:**
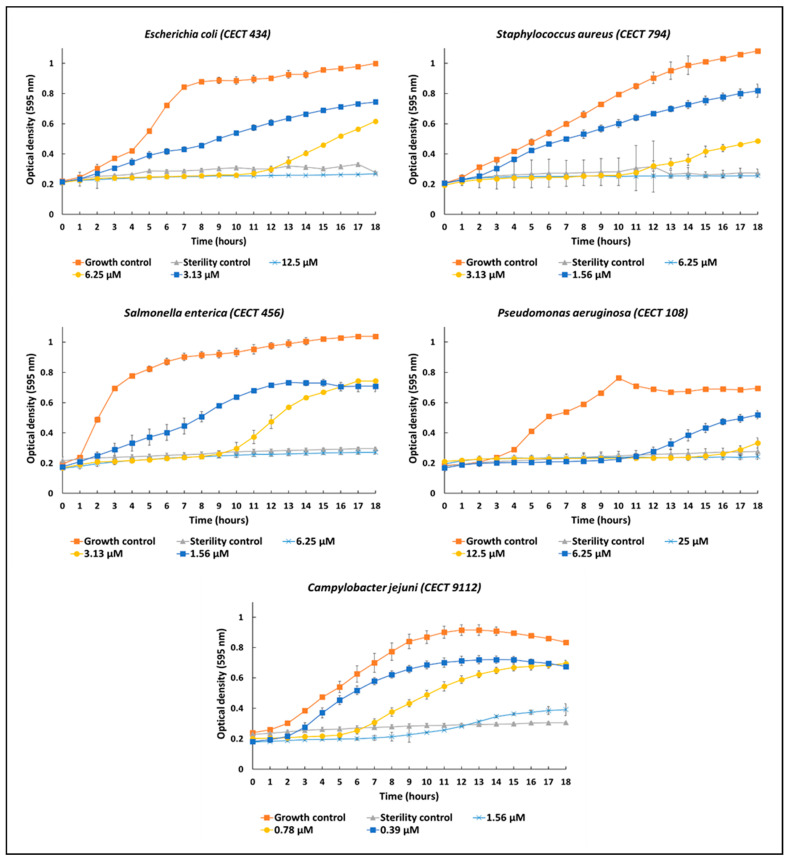
Kinetic studies of five microorganisms against different concentrations above and below the MIC of the synthetic peptide To-KL37 during 18 h of in vitro culture.

We next performed a kinetic analysis of the in vitro antimicrobial activity of the synthetic peptide To-KL37 (Figure 7). In general, when using one or two peptide dilutions above and below the MIC, the growth of the microorganisms *P. aeruginosa* and *C. jejuni* was inhibited during the first few hours (<10 h), thus suggesting the bacteriostatic activity of the peptide. On the other hand, the kinetic analysis of the peptide with the microorganisms *Staphylococcus aureus*, *Escherichia coli*, and *Salmonella enterica* could be inhibited during the first 10 h, suggesting that the synthetic peptide To-KL37 was bactericidal rather than bacteriostatic (Figure 7). 

In order to ascertain whether the synthetic peptide To-KL37 was bacteriostatic or bactericidal, we tested the growing capacity of the microorganism after being incubated with the peptide at the higher concentration of the MIC value. This colony counting assay allowed us to confirm the bactericidal effect of the peptides at their minimum inhibitory concentration, except for *P. aeruginosa* and *C. jejuni*, which could still grow on solid media after 24 h despite having been cultured in the presence of the peptide at its minimum inhibitory concentration. For these microorganisms, the activity of the peptide was classified as bacteriostatic (Table 7).

**Figure 8:**
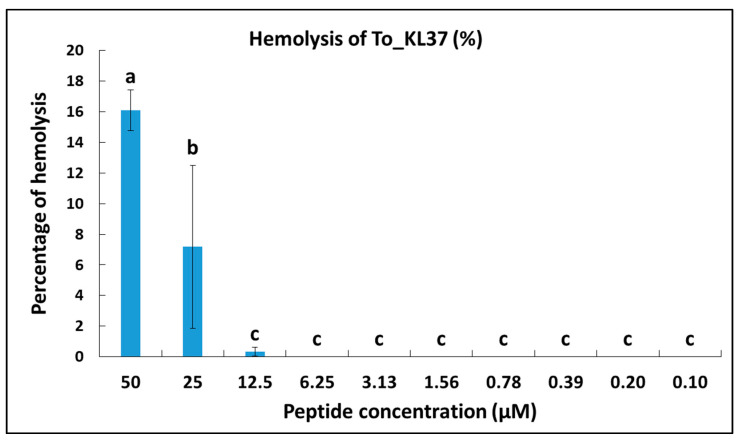
Percentage of hemolysis of human red blood cells in the presence of different concentrations of the synthetic peptide To-KL37, between 50 and 0.1 µM. ^a,b,c^ Means with different superscript letters differed significantly (*p* < 0.05), using one-way ANOVA and Tukey’s test.

Concerning the hemolytic activity of the synthetic peptide To-KL37, at concentrations below 25 µM we did not observe hemolysis to human erythrocytes. A lower percentage of hemolysis (7%) was observed when using the peptide at higher concentrations of 25 µM. The highest percentage of hemolysis for the synthetic peptide To-KL37 (16%) was found at the maximum concentration of 50 µM (Figure 8).

The active portion of most cathelicidins having the LPS-binding domain exert LPS-neutralizing activity inhibiting the inflammatory response of LPS, a constituent of the outer membrane of the cell walls of gram-negative bacteria [37]. Pathogen-associated chemical patterns, such as LPS, bind to pattern-recognition receptors, such as toll-like receptors, to activate innate immunity. The Toll-like receptor 4 (TLR4) also requires myeloid differentiation 2 (MD-2) for LPS recognition by its extracellular portion. LPS can fit into a hydrophobic cavity of MD-2, and this binding leads to homodimerization of the TLR4/MD-2 complex, which results in the activation of TLR4 downstream signaling. Direct complex formation between LPS and cathelicidins, including human LL-37, has now been proven to have a crucial role in inhibiting LPS binding to the receptor complex, hence suppressing immunological activation [38].

**Figure 9:**
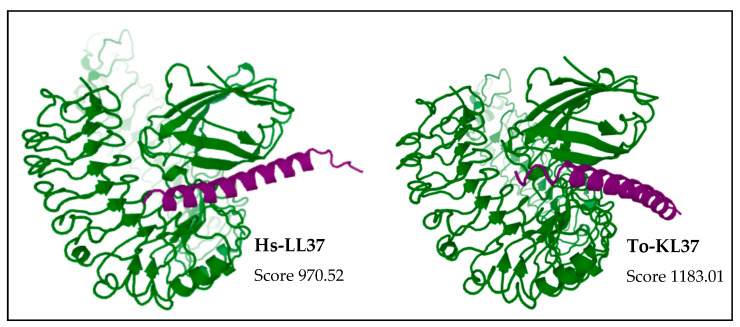
Molecular docking between the complex of murine TLR4/MD2 (PDB accession 2Z64) and the Alphafold model of the putative cathelicidin peptide of the Iberian mole (*Talpa occidentalis*). The human cathelicidin peptide Hs-LL37 was similarly used as a reference peptide. The analysis of the molecular interaction was performed by the GalaxyTongDock_A web server (https://galaxy.seoklab.org/index.html accessed on 9 May 2022). Purple protein corresponds to the peptide and green protein corresponds to the TLR4/MD2 complex. The final models were obtained by clustering similar model structures. They were ranked by the cluster size (or score when the interface option is used). The model with highest TongDock_A score was chosen for the analysis of the interaction. The GalaxyTongDock score has the same absolute value as the GalaxyTongDock energy but with the opposite sign).

The possible mechanism underlying the antimicrobial action of the synthetic peptide To-KL37 was investigated by molecular docking, taking the human cathelicidin Hs-LL-37 as a reference peptide for the potential interaction (Figure 9).

Molecular docking revealed that the peptide from the Iberian Mole To-KL37 attached to the TLR4/MD2 complex, and that the binding site was near the MD2 protein (Figure 9). The intricate docking structure of To-KL37 and MD2 reveals that To-KL37 is located at the entrance of the LPS-binding pocket in the MD2 molecule (Figure 9). This suggests that To-KL37 might bind to the TLR4/MD2 complex and overlaps the LPS-binding pocket in MD2, thereby inhibiting the binding of LPS to MD2 and the subsequent activation of signaling pathways. This result was consistent with the peptide of reference, the human cathelicidin Hs-LL37, which also could inhibit the binding of LPS to the TLR4/MD2 complex (Figure 9). The score for the interaction of the peptide from the Iberian mole (1183.01) was higher than the score found for the interaction of human Hs-LL37 (970.52), which means that the interaction of the Iberian Mole To-KL37 peptide with the TLR4/MD2 complex required less energy than the human Hs-LL37 peptide.

## 4. Discussion

We believe that nature contains an almost unlimited number of peptide therapeutics that have yet to be pharmacologically characterized [39]. It has been suggested that the vast diversity of cationic peptides originated in each host organism from their antibacterial role to fight pathogenic microbes [40]. Particularly, organisms inhabiting settings surrounded by microorganisms are a rich source of antimicrobials that can likely offer us superior templates for the development of new antimicrobial drugs to combat the challenging medical disorders that prevail today [41,42]. Because of their conservation of several rudimentary traits, insectivore animals have been considered to be closely related to the original mammalian line for more than a century. This has made insectivores a particularly interesting group in the study of mammalian evolution. They are well-adapted to a wide range of settings, including urbanized regions, where they serve as a link between the natural and human worlds through the frequent mobility of these animals [43,44]. For example, tick species depend on small mammals for their survival and growth, including hard ticks and *Ixodes ricinus* in their immature stages. Small mammals also serve as possible reservoirs for microorganisms, such as *Anaplasma phagocytophilum* [45,46].

In the innate immune response of mammalian organisms against bacterial infection, the cathelicidin family of endogenous antimicrobial peptides plays an important role [47,48]. The promise of cathelicidin-derived antimicrobial peptides has been recognized, owing to their higher bactericidal efficacy, when compared to chemical antibiotics, as well as their exceptional mechanisms of action, preventing the development of drug resistance [49,50].

Using the NCBI genome database, we were able to identify and characterize the Iberian mole cathelicidin. The annotation of the sequences as Iberian mole is an automated computational procedure performed by the NCBI using the Gnomon gene prediction algorithm. The presence of a conserved cathelin-like domain was used to determine the nature of the cathelicidin family. The bioinformatic analysis of cathelicidins seems to be effective in finding the biologically active region of the protein, allowing the sequence of the active peptide to be obtained, as well as the prediction of the peptides most likely to be active [36]. In our analysis, the common shrew (*Sorex araneus*) was the only *Eulipotyphla* cathelicidin from which no biological activity was predicted, being the one with the smallest a-helix region, with no positive net charge, less hydrophobicity, and a distinct mode of interaction with simulated membranes. On the other hand, our in silico analysis was consistent with the net charge and secondary structure of each peptide, as those with a lower net charge and/or lower helicity exhibited a lower degree of contact with simulated membranes. These and other techniques predicted the antibacterial and antiviral activities of the putative peptides. The interaction between cationic peptides and negatively charged lipid membranes of microbes promotes accurate, simultaneous adhesion and anchoring, hence facilitating the disruption of the microbial membrane [51]. As a part of our research, we simulated the membrane interaction of the predicted active peptides from the *Eulipotyphla* cathelicidins. Cathelicidins prefer negatively charged prokaryotic membranes with high electrical potential gradients as a requirement for cell entry or direct destruction of the bacterial cell membrane. Altering the secondary and tertiary structures of the peptide could cause a shift in the direction in which it is aligned perpendicular to the membrane, which would result in the peptide becoming embedded in the lipid bilayer and producing transmembrane pores.

Considering the high cost of peptide synthesis and taking into account the biochemical, structural, and in silico prediction, the Iberian mole-derived cathelicidin peptide (To-KL37) was selected for chemical synthesis to perform the in vitro experiments. Like the other peptides of the available cathelicidins of the order *Eulipotyphla,* Sa-NN35 could be discarded for antimicrobial testing because its biochemical properties and structure differed significantly from those of the others. However, it is possible that this peptide has other activities, such as immunomodulatory, cellular signaling, antitumor, or healing activity [52]. Future in vitro assays could take advantage of the antibacterial potential of peptides Ee-RL42 and Cc-KL41. A limitation of our study was the larger sequence of these peptides, which drives up the cost of producing the synthetic peptides.

The antimicrobial activity of the putative active peptide of the Iberian Mole To-KL37 was observed in vitro at very low concentrations. In general, the growth of the microorganisms *P. aeruginosa* and *C. jejuni* was inhibited when using one or two peptide dilutions above and below the MIC during the first few hours (<10 h), thus suggesting the bacteriostatic activity of the peptide. On the other hand, the kinetic analysis of the peptide To-KL37 with the microorganisms *Staphylococcus aureus*, *Escherichia coli*, and *Salmonella enterica* could be inhibited during the first 10 h, suggesting that the synthetic peptide To-KL37 was bactericidal rather than bacteriostatic. To-KL37 was active against different microorganisms with a minimum inhibitory concentration between 25 µM (with *S. aureus*) and 1.56 µM (with *C. jejuni*). These low concentrations, together with its low percentage of hemolysis, make the To-KL37 a peptide with apparently low toxicity and good antimicrobial activity. Cc_KL41 has been reported to have antimicrobial activity (MIC) against *Bacillus cereus* (0.8–0.4 µM), *Enterococcus faecalis* (3.1–1.6 µM), *Candida albicans* (12.5–6.3 µM), *Pseudomona corrugata* (25–12.5 µM) and *E. coli* (1.5–0.8 µM) [53].

The Hs-LL37 peptide has broad-spectrum antibacterial activity against Gram-negative and Gram-positive bacteria, fungi, and even protozoa. This is consistent with our findings that cathelicidin-related peptides, such as To-KL37, have broad antibacterial activity [54,55,56].

By molecular docking, we also investigated the antimicrobial peptide To-KL37 of *Talpa occidentalis* and provided structural and bioinformatics evidence to support its LPS-mediated immunomodulatory activity. Although other cytotoxicity tests are still necessary, this peptide might have a good potential for its application in biomedicine, since it has bactericidal/bacteriostatic activity against all microorganisms studied. *C. jejuni* is a zoonotic agent. The apparent activity against this bacterium could be related to the lifestyle of these animals and their close relationship with soil microorganisms [48,57]. The use of anthropogenic landscapes by wildlife may increase exposure and infection with *C. jejuni*. While some *Campylobacter* species are pathogenic to animals and have been associated with infertility and abortions, it is important to know that *C. jejuni* and *C. coli* are considered low pathogenic and nonpathogenic to animals, respectively [58]. To better understand the epidemiology of *Campylobacter*, it is important to evaluate *Campylobacter* transmission in animal reservoirs [59]. It should be interesting to investigate the presence of these enteric microorganisms in the Iberian mole, *Talpa occindetalis.* It is possible that the cathelicidin antimicrobial peptide To-KL37 plays an important role in the low pathogenicity of *C. jejuni* in these species.

## 5. Conclusions

The presence of cathelicidins in a wide variety of organisms is evidence supporting their significance. Their variability is a crucial resource in the development of new therapies owing to the various microbiological problems these species have to face [60]. Learning more about insectivores’ antimicrobial defenses can help scientists build better strategies to predict infection spread to humans. Based on the results of our preliminary in silico research, we anticipate that the cathelicidin peptides found in the Iberian mole will have antibacterial properties. We presented data to confirm the antibacterial effect of the predicted cathelicidin from *Talpa occidentalis* by comparing its properties to those of the human LL-37 protein. The study of how insectivore mammals use antimicrobial peptides in their innate immune systems could provide novel, strong antimicrobial peptides for potential therapeutic development.

## Figures and Tables

**Table 1:** Amino acid sequence of the putative antimicrobial peptide for the annotated cathelicidin sequences (accessed on 9 May 2022) of mammals of the *Eulipotyphla* order, including *Talpa occidentalis*. Shaded residues correspond to the alpha helix portion of the peptide according to the models predicted with the Alphafold server. Residues in red represent the LPS binding domain of CAP18 (identified by its similarity with pfam12153).

Specie	Accession	Name	Predicted Peptide Sequence
*Sorex araneus*	XP_004615204	Sa-NN35	NNDINLKVNIAQNRNELERQDPNICRRKRHKTFPM
*Erinaceus europaeus*	XP_007533667	Ee-RL42	RLFGRLRDLIKKGTQKIGRKLRKVGQQIKDFIRNLRPREEDS
*Condylura cristata*	XP_012590677	Cc-KL41	KLFGKVGDFLKRGGQKIGEKIEKIGKRIKDFFQNLKPREEA
*Talpa occidentalis*	XP_037384309	To-KL37	KLFGKVGNLLQKGWQKIKNIGRRIKDFFRNIRPMQEA
*Homo sapiens*	PDB: 5NNT_A	Hs-LL-37	LLGDFFRKSKEKIGKEFKRIVQRIKDFLRNLVPRTES

**Table 2:** Helix properties calculation of putative antimicrobial peptides from *Eulipotyphla* order, including *Talpa occidentalis*, obtained by the bioinformatic tool “Heliquest” (https://heliquest.ipmc.cnrs.fr/cgi-bin/ComputParams.py), accessed on 9 May 2022.

Peptide	Hydrophobicity <H>	Net Charge z	Hydrophobic Moment <µH>	Hydrophobic Face
Ee-RL42	0.205	9	0.668	LVILIIFL
Sa-NN35	0.002	0	0.143	LLI
Cc-KL41	0.187	5	0.627	VIILIFI
To-KL37	0.338	7	0.683	LIGIVIILFFGW
Hs-LL37	0.174	6	0.693	LIILFIF

**Table 3:** Models and parameters obtained from the server Orientation of Proteins in Membranes (https://opm.phar.umich.edu/ accessed on 9 May 2022). A computational approach for optimizing the spatial arrangement of protein structures in lipid bilayers.

Peptide	Position of Embedded Residues	Tilt Angle	Image in Membrane
Ee-RL42	1–7,9–10,14,17,21,28,31–32,35,37	86	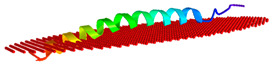
Sa-NN35	33–35	67	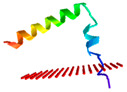
Cc-KL41	1–7,9–10,17,21,32,38,40–41	84	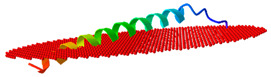
To-KL37	2–3,6,9–10,14,17,20,24,27–28,31–32,34–35,37	89	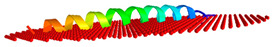
Hs-LL37	1–7,9–10,13,17,20–21,24,27–28,31–33	85	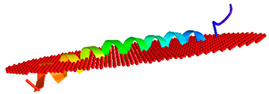

**Table 4:** Bioinformatic prediction of the biological activity for the putative peptides from the *Eulipotyphla* order, including *Talpa occidentalis*, and using human cathelicidin Hs-LL37 as a reference. Values computed from different servers represent scores for each biological activity within the following numerical range: antimicrobial (0–1), antiviral (0–100%).

Peptide	Antimicrobial	Antifungal	Antiviral	Anticancer
Ee-RL42	0.90	No	73.58%	No
Sa-NN35	0.54	No	No	No
Cc-KL41	0.80	No	76.77%	0.98
To-KL37	0.95	No	80.66%	0.89
Hs-LL37	0.76	No	78.13%	0.99

**Table 5:** Bioinformatic prediction of the biological activity against specific microorganisms. The Bioinformatic tool (database of antimicrobial activity and structure of peptides, DBAASP) predicted the susceptibility of microorganisms and human erythrocytes to the putative peptides from the *Eulipotyphla* order, including *Talpa occidentalis*, and using human cathelicidin Hs-LL37 as a reference.

Peptide	*Escherichia* *coli* ATCC 25922	*Pseudomonas* *aeruginosa* ATCC 27853	*Klebsiella* *pneumoniae* *	*Staphylococcus aureus* ATCC 25923	*Bacilus* *subtilis* *	Hemolytic
Ee-RL42	Active	Active	Not Active	Not Active	Not Active	Not Active
Sa-NN35	Not active	Not active	Not active	Not active	Not active	Not active
Cc-KL41	Active	Active	Not Active	Not Active	Not Active	Active
To-KL37	Active	Active	Active	Active	Active	Not Active
Hs-LL37	Active	Active	Not Active	Not Active	Not Active	Not Active

* Specific strain not available in the bioinformatics tool.

**Table 6:** Minimum inhibitory concentration ranges of the synthetic peptide To-KL37 against several microorganisms.

Microorganisms	MIC Ranges (µM)
*Staphylococcus aureus* (CECT 794)	6.25–3.13
*Escherichia coli* (CECT 434)	12.5–6.25
*Salmonella enterica* (CECT 456)	6.25–3.13
*Pseudomonas aeruginosa* (CECT 108)	25–12.5
*Campylobacter jejuni* (CECT 9112)	1.56–0.78

**Table 7:** In vitro test for the detection of the growth of microorganisms after 18 h in the presence of the synthetic peptide at its higher value of the MIC concentration obtained for the microorganisms *Escherichia coli* (CECT 434), *Staphylococcus aureus* (CECT 794), *Salmonella enterica* (CECT 456), *Pseudomonas aeruginosa* (CECT 108), and *Campylobacter jejuni* (CECT 9112).

Microorganism	*E. coli*	*S. aureus*	*S. enterica*	*P. aeruginosa*	*C. jejuni*
Growth after 24 h	Negative	Negative	Negative	Positive	Positive
Peptide activity	Bactericide	Bactericide	Bactericide	Bacteriostatic	Bacteriostatic

## Data Availability

Not applicable.
